# Randomized Clinical Trial Comparing Quadratus Lumborum Block and Intrathecal Morphine for Postcesarean Analgesia

**DOI:** 10.1055/s-0042-1759728

**Published:** 2022-12-29

**Authors:** Karoline Moura de Araújo, Leonardo Henrique Cunha Ferraro, Sue Yasaki Sun, Rosiane Mattar

**Affiliations:** 1 Escola Paulista de Medicina, Universidade Federal de São Paulo, São Paulo, SP, Brazil

**Keywords:** analgesia, cesarean section, morphine, spinal anesthesia, anestesia, obstetrical, analgesia, cesariana, morfina, anestesia espinhal, anestesia obstétrica

## Abstract

**Objective** To compare the efficacy of quadratus lumborum (QL) block and intrathecal morphine (M) for postcesarean delivery analgesia.

**Methods** Thirty-one pregnant women with ≥ 37 weeks of gestation submitted to elective cesarean section were included in the study. They were randomly allocated to either the QL group (12.5 mg 0.5% bupivacaine for spinal anesthesia and 0.3 ml/kg 0.2% bupivacaine for QL block) or the M group (12.5 mg bupivacaine 0.5% and 100 mcg of morphine in spinal anesthesia). The visual analog scale of pain, consumption of morphine and tramadol for pain relief in 48 hours, and side effects were recorded.

**Results** Median pain score and/or pain variation were higher in the morphine group than in the QL group ( *p* = 0.02). There was no significant difference in the consumption of morphine or tramadol between groups over time. Side effects such as pruritus, nausea, and vomiting were observed only in the morphine group.

**Conclusion** Quadratus lumborum block and intrathecal morphine are effective for analgesia after cesarean section. Patients undergoing QL block had lower postoperative pain scores without the undesirable side effects of opioids such as nausea, vomiting, and pruritus.

## Resumo

**Objetivo** Comparar a eficácia do bloqueio do quadrado lombar (QL) e da morfina intratecal (M) na analgesia pós-cesariana.

**Métodos** Trinta e uma gestantes com ≥ 37 semanas de gestação submetidas a cesariana eletiva foram incluídas no estudo. Eles foram alocados aleatoriamente no grupo QL (12,5 mg de bupivacaína a 0,5% para raquianestesia e 0,3 ml/kg de bupivacaína a 0,2% para bloqueio de QL) ou no grupo M (12,5 mg de bupivacaína a 0,5% e 100 mcg de morfina na raquianestesia). A escala visual analógica de dor, consumo de morfina e tramadol para alívio da dor em 48 horas e efeitos colaterais foram registrados.

**Resultados** A mediana do escore de dor e/ou variação da dor foi maior no grupo morfina do que no grupo QL ( *p* = 0,02). Não houve diferença significativa no consumo de morfina ou tramadol entre os grupos ao longo do tempo. Efeitos colaterais como prurido, náuseas e vômitos foram observados apenas no grupo morfina.

**Conclusão** O bloqueio QL e a morfina intratecal são eficazes para analgesia após cesariana. Os pacientes submetidos ao bloqueio do QL apresentaram menores escores de dor pós-operatória sem os efeitos colaterais indesejáveis dos opioides, como náuseas, vômitos e prurido.

## Introduction

Cesarean section is the most frequently performed surgical procedure in obstetrics. 1 The number of cesarean deliveries has increased in recent years, accounting for ∼ 21% of births worldwide. 2

During the postoperative period, ∼ 1 of 5 women experience acute pain. 3 The effective management of postoperative pain is highly important as it facilitates early recovery, ambulation, and breastfeeding, allowing mothers to provide better care to their newborns. 4 5 Moreover, it helps preventing venous thromboembolism 6 and respiratory complications, decreasing hospital stay. 7 In addition to these benefits, an adequate management of acute postcesarean pain is associated with a 3-fold decrease in the risk of postpartum depression 8 and development of chronic pelvic pain. 9 10

Morphine (M) has been widely used for postoperative pain relief, due to its favorable pharmacokinetic profile, ease of administration during spinal block and low cost. 11 12 However, the use of opioids is associated with undesirable side effects such as nausea, vomiting, pruritus, and urinary retention, which can reduce patient satisfaction. Furthermore, the fact that M may produce severe maternal respiratory depression underscores the importance of investigating alternative opioid-free analgesia approaches. 13 14

Another opioid used for postoperative pain relief is Tramadol. This is a synthetic 4-phenyl-piperidine analogue of codeine with a dual mechanism of action. It stimulates µ receptors and, to a lesser extent, δ and Ϗ receptors. Like tricyclic antidepressants, tramadol also activates spinal pain inhibition by decreasing the reuptake of norepinephrine and serotonin. 15

The blockade of peripheral nerves for analgesia of the abdominal wall after surgery has become more frequent, especially with the development of ultrasound technology. 16 17

Quadratus lumborum (QL) block is a technique used to inject local anesthetic in the posterior abdominal wall around the quadratus lumborum muscle to anesthetize the thoracolumbar nerves. 18 It can provide somatic as well as visceral analgesia due to its paravertebral spread. 19 20 According to a systematic review by Jin et al., 21 QL block significantly reduces opioid requirement in cesarean delivery and in renal surgery. Similarly, Graça et al. 22 have reported a reduction in postoperative opioid consumption with the use of QL block in laparoscopic nephrectomy, while Zhu et al. 23 have found that anterior QL block significantly alleviated pain in patients undergoing open liver resection.

The objective of the present study was to compare the efficacy of QL block and intrathecal M for postcesarean delivery analgesia by measuring M/tramadol consumption during the first 48 hours after surgery.

## Methods

The present randomized clinical trial was registered at the Brazilian Clinical Trials Registry (RBR-5RHP9J) and was approved by the Research Ethics Committee of the Universidade Federal de São Paulo (UNIFESP, in the Portuguese acronym) (CEP/UNIFESP.CAAE:83549817.3.0000.5505), was conducted at the Hospital São Paulo, UNIFESP.

The inclusion criteria were as follows: pregnant women > 18 years old with gestational age of at least 37 weeks, normal singleton pregnancy, physical status classified according to the American Society of Anesthesiologists as ASAII (mild systemic disease without functional limitations), and ASA III (mild systemic disease with functional limitations), elective cesarean section performed under spinal anesthesia at the Obstetric Center of the Faculdade Paulista de Medicina between June 2019 and December 2019.

Exclusion criteria: inability to understand or provide a verbal self-report of pain on a scale, congenital or acquired coagulation disorders, allergy to local anesthetics, anatomical disorders of the spine leading to neuraxial block failure, BMI > 35 kg/m ^2^ , and local infection.

Initially, we defined that the sample size would be for an established period of time. However, during our study, Salama 24 published a study similar to ours.

Considering total postoperative morphine consumption as significantly lower in the QL block group than in the M group, as reported by Salama, 24 and assuming a statistical power of 80% at a significance level of 0.5%, a sample size of 4 participants in each group was estimated to be enough to compare efficacy between QL block and intrathecal morphine for postcesarean delivery analgesia on the basis of total postoperative M consumption. Thus, we used a secondary outcome obtained in the study by Salama, 24 total postoperative morphine consumption, to calculate our sample size.

However, at that time, we had already collected more patients: 15 to the QL block group and 16 to the M group. So, we decided to present data from all patients studied.

Eligible parturients were invited to participate in the study during the preanesthetic visit. Patients who agreed to participate in the research signed an informed consent form.

Study participants were randomly allocated into two groups: Group M (spinal anesthesia with bupivacaine and M), and Group QL (spinal anesthesia with bupivacaine + QL block). The randomization was performed using software available at http://github.com/Gear61/Random-Number-Generator (v. 2015). This software generated a numerical sequence of 1 or 2. The patient who was randomized with the number 1 would belong to the M group and the one randomized with the number 2 would belong to the QL group. Before the procedure, the patients did not know which group they belonged to.

The study procedures were performed by two anesthesiologists. “Anesthesiologist One” (AO) conducted randomization, filled the syringes with the study medication, and performed the QL block. Anesthesiologist One is a specialist in regional anesthesia, with 5 years of experience in regional blocks. “Anesthesiologist Two” (AT) administered spinal anesthesia but did not know the volume of drugs that each group would receive during spinal anesthesia. Anesthesiologist Two was blind to patient allocation and performed the postoperative assessment.

Both groups received 12.5 mg of 0.5% hyperbaric bupivacaine for spinal anesthesia. To group M, 100 mcg of M, whose onset of action occurs in 60 minutes, was added to the syringe containing bupivacaine.

To group QL, bilateral QL block was performed by injecting 0.3 mL/kg of 0.2% bupivacaine on each side.

Spinal anesthesia was performed in the sitting position at vertebral levels L3-L4 or L4-L5, using a 27-gauge pencil point needle according to the standard hospital protocol. After surgery, AT left the operating room to return 1 hour later to reevaluate the patient.

In the QL group, bilateral US-guided QL block was performed by AO using a Sonosite M-Turbo R System with HFL 38x, 5–8 MHz convex transducer (Sonosite, Bothell, WA, USA). With the patient in the lateral decubitus position, the transducer was placed at the anterosuperior iliac spine level and advanced cranially to visualize the three abdominal muscle layers. The external oblique muscle was followed laterally until its posterior border was visualized with the internal oblique muscle underneath, like a roof over the QL muscle. The transducer was directed downwards to identify the middle layer of the thoracolumbar fascia as a bright hyperechoic line.

After antisepsis of the anterior abdominal wall with alcoholic chlorhexidine, a 22G x 100 mm needle (AEq. 2250, BMD Group, Venice, Italy) was inserted in-plane from the anteromedial to the poster direction, at an angle of 45 degrees to the skin for the injection of bupivacaine. Thus, all patients received type 2 QL block. The performance of QL block took ∼ 5 minutes.

Postoperative analgesia for all patients consisted of dipyrone 1 g every 6 hours, and ketoprofen 100 mg every 12 hours, both intravenous. We followed the postoperative rescue analgesia protocol adopted at the hospital: at any time during the postoperative period, patients could request rescue medication for pain relief. If the patient reported a moderate pain score (4–6), tramadol 100 mg was administered intravenously every 6 hours. If there was no improvement in pain after tramadol or if the patient reported severe pain score (7–10), the rescue medication used was M 4 mg, intravenously, every 6 hours.

The AD evaluated the parameters, described below, in all patients at predetermined intervals after surgery (1, 2, 4, 6, 12, 24, and 48 hours); with the main outcome being the consumption of M/tramadol. Morphine/tramadol consumption was measured in milligrams (mg), heart rate in beats per minute (bpm), oxygen saturation in percentage of oxygen carried by the blood (%), noninvasive blood pressure in millimeters of mercury (mmHg). Pain scores were evaluated using the visual analogue pain scale, whose values range from 0 to 10; zero means total absence of pain and 10 the maximum level of pain bearable by the patient, with a score of 1 to 3 considered as mild, moderate from 4 to 6, and severe from 7 to 10. The sedation level was measured as follows: grade 1 = anxious and agitated patient; grade 2 = cooperative, oriented and calm patient; grade 3 = sleepy patient and attentive to commands; grade 4 =patient sleeping, responds quickly to vigorous sound stimulus, grade 5 = patient sleeping, responds slowly to vigorous sound stimulus, and grade 6 = patient sleeping, no response. Pruritus was evaluated as follows: grade 0 = absent, grade 1 = mild, grade 2 = moderate, grade 3 = severe. Nausea was measured as follows: grade 0 = absent, grade 1 = mild, grade 2 = moderate, grade 3 = severe or vomiting. In addition to these parameters, the presence or absence of residual block and other complications was evaluated.

Quantitative variables were compared using the parametric Student *t* -test or the nonparametric Mann-Whitney test. For the comparison of qualitative variables between groups, the chi-squared test, the Fisher exact test or the likelihood ratio test were performed. To compare quantitative variables between groups over time, analysis of variance (ANOVA) with repeated measures or repeated measures ANOVA with rank transform were used. To compare qualitative variables between groups over time, the generalized estimating equation (GEE) model was used. Significance was set at 5% ( *p* < 0.05).

## Results

**Fig. 1:**
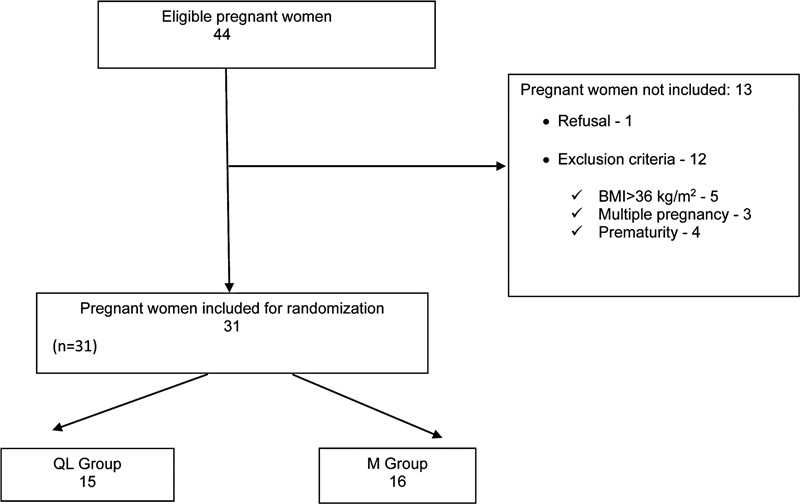
Study flow diagram.

As shown in the diagram below, 44 patients were invited to participate in the study; 13 were not included in the study for the following reasons: 1 refused to participate, 12 met the exclusion criteria; 5 had BMI > 36 kg/m2, 4 were < 37 weeks pregnant, and 3 were with multiple pregnancies. Thus, the study population consisted of 31 parturients. Of these, 15 were assigned to the QL group, and 16 to the M group ( Fig. 1 ).

As shown in Table 1 , there was no significant difference between the groups regarding age, BMI, and number of previous cesarean deliveries. Only one patient in the QL group was classified as ASA III, all others in both groups were ASA II, showing the homogeneity of the samples.

**Table 1:** Clinical and demographic characteristics of study patients

Variables by group	QL	M	*p-value*
**Age (Years old)**			
Mean (SD)	33.5 (6.7)	31.1 (6.4)	0.293 ^(**)^
Median (Minimum–Maximum)	37 (19–41)	31 (18–41)	
Total	15	16	
**ASA −** ***n*** **(%)**			
II	14 (93.3)	16 (100)	not calculated
III	1 (6.7)	0 (0)	
Total	15 (100)	16 (100)	
**BMI**			
Mean (SD)	30.8 (3.9)	30.2 (4.8)	0.669 ^(*)^
Median (Minimum–Maximum)	30 (24.5–36)	30.3 (21.6–36)	
Total	15	16	
**Previous cesarean delivery −** ***n*** **(%)**			
No	5 (33.3)	10 (62.5)	
Yes	10 (66.7)	6 (37.5)	0.104 ^(#)^
Total	15 (100)	16 (100)	
**Previous cesarean delivery**			
Mean (SD)	1.2 (1.15)	0.81 (1.17)	0.279 ^(**)^
Median (Minimum–Maximum)	1 (0–4)	0 (0–3)	
Total	15	16	

Abbreviation: SD. standard deviation.

(*) Parametric t-Student test; (**) Mann-Whitney nonparametric test; (#) Chi-squared test.

Table 2 shows that most patients in both groups received neither morphine nor tramadol over 48 hours after surgery. Morphine consumption ( Table1 ) was lower in the QL group (33.4%) compared with the M group (43.9%), but no statistical difference was reached. Moreover, there were twice as many patients who used M in group M compared with group QL at 12 hours and 24 hours after cesarean section. In the QL group, tramadol was used by 26.7% of the patients at 2, 6, and 12 hours and by 13.3% at 24 hours. In contrast, the use of tramadol in group M was higher at 24 hours (18.8%) than at 6 and 12 hours (6.3%). Nonetheless, no statistical difference was observed between groups. During the period in which they were evaluated, six patients used both medications: M and tramadol, all of which were in the M group.

**Table 2:** Opioid consumption and urinary retention between groups

Time	Consumption of tramadol			Consumption of morphine			Urinary retention	
**(Post-** **operative)**	** M** **group** ***n*** **(%)**	**QL group** ***n*** **(%)**	***p*** **a**	** M** ** group** ***n*** **(%)**	** QL** ** group** ***n*** **(%)**	***p*** **b**	**M group** ***n*** **(%)**	**QL group** ***n*** **(%)**
1 hour	1(6.25)	0	0.059	1(6.25)	1(6.66)	0.631	0	0
2 hours	2(12.5)	4(26.66)		1(6.25)	1(6.66)		0	0
4 hours	3(18.75)	0		1(6.25)	2(13.33)		1(6.25)	0
6 hours	1(6.25)	4(26.66)		1(6.25)	0		4(25)	0
12 hours	1(6.25)	4(26.66)		2(12.5)	1(6.66)		4(25)	0
24 hours	3(18.75)	2(13.33)		2(12.5)	1(6.66)		4(25)	1(6.66)
48 hours	0	1(6.66)		0	0		1(6.25)	0

Abbreviations: M group, morphine group. QL group: quadratus lumborum group.

a Generalized estimating equations model (GEE).

b Mann-Whitney nonparametric test.

**Fig. 2:**
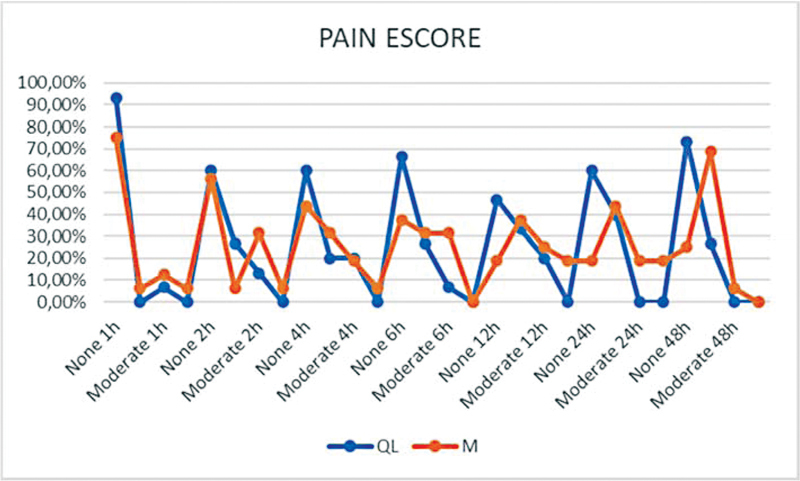
Distribution of pain score over time among patients in the quadratus lumborum and morphine groups. M: Morphine Group, QL: Quadratus Lumborum Group. p-value = 0.002. Model of Analysis of Variance (ANOVA).

As shown in Fig. 2 , the pain scores significantly differed between groups ( *p* = 0.002) independently of time ( *p* = 0.162). Median pain scores and/or pain variation, despite being higher in group M than in group QL.

Pruritus was not observed in any of the QL patients. On the other hand, in group M, pruritus was present in half of the women after 4 hours, in nearly 70% after 12 hours, and was still present in 12.6% of them 48 hours after surgery. Nausea was not seen in any of the women in the QL group but was present from the first hour to 24 hours postoperatively in 12 to 20% of the participants in group M. Urinary retention occurred in only 1 woman in the QL group, whereas in the M group it occurred in 25% of the patients from 6 hours to 24 hours after cesarean section. No patient had respiratory depression in any of the groups. Residual block was present in only one patient in the QL group. Heart rate, respiratory rate, systolic blood pressure, diastolic blood pressure, oxygen saturation, and sedation score did not significantly differ between groups. All patients in the study had a grade 1 sedation score, that is, they remained cooperative, oriented, and calm.

## Discussion

The present study demonstrated that QL block and intrathecal M injection are effective in providing postoperative analgesia in patients undergoing cesarean section as there was no significant difference in opioid consumption in 48 hours. In the QL group, pain scores were significantly lower and side effects such as pruritus, nausea, and vomiting were not observed.

Our results show that QL block and intrathecal morphine can effectively relief postoperative pain. Indeed, 86% of the patients in both groups did not require the administration of opioids.

Quadratus lumborum block type 2 was the technique of choice in the present study because it was demonstrated by Blanco et al. 25 to be superior to transversus abdominis plane blocks (TAP blocks) in providing postoperative analgesia. However, a study by Kang et al. 26 comparing the effects of epidural M and major QL block approaches showed that the combination of QL block type 2 and 3 can provide superior postcesarean analgesic effect.

Morphine and tramadol consumption did not differ between the study groups ( Table 2 ). Morphine consumption 6 hours after cesarean section was lower in the QL group, suggesting that QL block has a longer lasting effect. However, this finding did not reach statistical significance, in opposition to Salama, 24 who observed a significant lower morphine consumption in the QL group. Our results also differ from those of Kang et al., 26 who reported significantly higher morphine consumption with QL block compared with peridural morphine. These diverging results may be explained by differences in the local anesthetic dose and volume used. While higher concentrations (0.375% ropivacaine) were used by Salama, 24 the participants of the present study received the same concentration used by Kang et al. 26 (0.2% ropivacaine) and Blanco et al. 19 (0.2% bupivacaine). Furthermore, anesthetic volumes also differed; Salama 24 used 24 ml on each side of the block, whereas the volume adopted in the present study, as well as by Blanco et al. 19 was 0.3 ml/kg, and that used by Kang et al. 26 was 30 ml on each side.

Pain scores over 48 hours among our patients were significantly lower in the QL group ( Fig. 2 ), indicating that QL block was effective as an anesthetic technique. The spreading of QL block into the paravertebral space 24 and into thoracic and lumbar sympathetic nerves 27 seems to be the major mechanism of action of this anesthetic approach and might explain the lower pain scores found in our study.

Pain intensity and elective cesarean section have been associated with a negative birth experience, 28 and are related to post-traumatic stress symptoms, and postpartum depressive symptoms. 29 Within this framework, QL block stands as an effective alternative, given that it not only provides analgesia but is also free of undesirable side effects that could render the experience of childbearing more negative.

The incidence of pruritus, nausea, and vomiting in the postoperative period was higher in the M group. As a matter of fact, these symptoms were not seen in the QL group.

Urinary retention was more frequent in the M group than in the QL group, which had only one patient with this symptom. However, this difference was not statistically significant.

No case of respiratory depression was observed in any of the study participants.

Residual block was seen in only one patient of the QL group up to 12 hours after cesarean section. Kang et al. 26 also described this event in two patients undergoing QL block. It is possible that a posterior dispersion of the local anesthetic occurred, and therefore the QL block behaved as type 3, a complication that has been previously described. 30

Hemodynamic parameters were similar in both study groups, which did not differ regarding heart rate, respiratory rate, systolic and diastolic blood pressure, and oxygen saturation.

It is noteworthy that the groups of patients herein investigated were homogeneous, and that the same investigator performed all blocks. However, the study had some limitations. Obese patients with BMI ≥ 35 kg/m ^2^ were not included. It was not possible to install a patient-controlled analgesia pump, as is done in large centers, due to the infrastructure of the institution, so we chose to use the rescue analgesia protocol adopted in our hospital, which includes the use of tramadol for moderate pain scores. This setback did not affect the progress of the research or the results we arrived at, allowing us to proceed with the research.

## Conclusion

The QL block can be seen as a valuable option for those patients with a previous history of nausea, vomiting, and itching. Perhaps performing the quadratus lumborum block with a greater volume and concentration of local anesthetic can provide analgesia for a period longer than 48 hours. In brief, both QL block and intrathecal M were demonstrated to be effective for postcesarean section analgesia. Nonetheless, QL block seemed to be more advantageous, given that it was associated with lower postoperative pain scores and absence of pruritus, nausea, and vomiting.
